# Malignant Extra-adrenal Paraganglioma of the Bladder: A Rare Clinical Entity

**DOI:** 10.7759/cureus.107388

**Published:** 2026-04-20

**Authors:** Venkata Ramana Korrapati, Lakshmi Kandhan, Chandru T, Natarajan Kumaresan, Rubina Singh

**Affiliations:** 1 Urology, Sri Ramachandra Institute of Higher Education and Research, Chennai, IND; 2 Urology and Renal Transplantation, Sri Ramachandra Institute of Higher Education and Research, Chennai, IND

**Keywords:** bladder paraganglioma, hematuria, malignant paraganglioma, neuroendocrine tumor, urinary normetanephrines

## Abstract

Bladder paragangliomas are uncommon extra-adrenal neuroendocrine tumors that arise from chromaffin tissue within the bladder wall. Clinical presentation varies from hematuria to catecholamine-related paroxysms, and malignant transformation, although uncommon, carries significant morbidity.

We report a 45-year-old woman who presented with painless hematuria. Contrast-enhanced computed tomography (CECT) revealed a 3.6×3 cm lobulated, enhancing posterior bladder wall mass. Transurethral resection of the bladder tumor (TURBT) was performed, and histopathology confirmed paraganglioma. The patient was asymptomatic for six months, when she re-presented with recurrent hematuria and palpitations. Urinary normetanephrines were markedly elevated (3239 μg/24 h; normal 119-451 μg/24 h). ^68^Ga-DOTANOC positron emission tomography (PET)/CT revealed recurrence with right external iliac nodal metastasis. Following α- and β-blockade, she underwent anterior pelvic exenteration with an ileal conduit. Intraoperative hypertensive crisis (blood pressure (BP) 224/120 mmHg) was managed with nitroglycerin infusion. Histopathology revealed malignant paraganglioma infiltrating perivesical fat (pT3b) with nodal metastasis (pN1). The tumor demonstrated comedo-type necrosis, >250 cells/HPF, atypical mitoses, and Ki-67 index of 40%. The Grading of Adrenal Pheochromocytoma and Paraganglioma (GAPP) score was 9, indicating poorly differentiated high-risk disease. Immunohistochemistry was positive for chromogranin, synaptophysin, S100, and INSM1. Margins were negative.

Bladder paragangliomas are rare but clinically significant due to their malignant potential. Radical surgery with meticulous perioperative hemodynamic control is the cornerstone in recurrent/metastatic disease. Lifelong follow-up with imaging and biochemical surveillance is mandatory.

## Introduction

Paragangliomas are rare extra-adrenal neuroendocrine tumors that arise from the paraganglionic tissue of the sympathetic and parasympathetic nervous systems. They may be functional, secreting catecholamines, or non-functional, and their clinical manifestations vary with tumor location and secretory activity [1].

Although most paragangliomas occur in the head, neck, and retroperitoneum, the urinary bladder is an exceedingly rare site, accounting for <0.05% of all bladder tumors. Since the first description in 1953, fewer than 250 cases have been reported worldwide. While most bladder paragangliomas are benign, malignant behavior is defined only by local invasion or metastasis, as histological features alone cannot predict aggressive potential [2].

Clinically, hematuria is the most frequent symptom, seen in more than half of patients, while functional tumors may produce adrenergic crises such as paroxysmal hypertension, palpitations, and micturition-induced syncope. These features often overlap with urothelial carcinoma or essential hypertension, contributing to delayed or missed diagnoses [3].

Radiologically, bladder paragangliomas usually present as avidly enhancing masses on computed tomography (CT) or magnetic resonance imaging (MRI); in some cases, a "light-bulb bright" appearance is noted on T2-weighted MRI. Functional imaging with ^68^Ga-DOTANOC positron emission tomography (PET)/CT has emerged as the most sensitive modality for localizing primary lesions, detecting recurrence, and identifying metastatic spread [4].

Histopathologically, these tumors display the classic zellballen architecture, with chief cell nests surrounded by sustentacular cells. Immunohistochemistry is typically positive for chromogranin, synaptophysin, and S100. Prognostic indices such as the Grading of Adrenal Pheochromocytoma and Paraganglioma (GAPP) score and Ki-67 index aid risk stratification, although malignant behavior can only be confirmed by metastasis. Recurrence has been reported in up to 15-30% of cases, and international guidelines recommend lifelong biochemical and imaging surveillance following surgical resection [5].

In this context, reporting malignant bladder paraganglioma with nodal metastasis is valuable, as such cases remain exceptionally rare and highlight diagnostic, therapeutic, and perioperative challenges.

## Case presentation

A 45-year-old woman, previously healthy, presented with one month of painless gross hematuria. She had no history of hypertension, palpitations, headaches, or micturition-induced syncope, and physical examination was unremarkable. Contrast-enhanced CT (CECT) of the abdomen and pelvis demonstrated a 3.6×3 cm arterial-enhancing lobulated intraluminal mass arising from the posterior bladder wall. Based on imaging, a bladder neoplasm was suspected, and she underwent transurethral resection of the bladder tumor (TURBT). Histopathology revealed a paraganglioma invading the lamina propria, with tumor cells arranged in a zellballen pattern and immunopositivity for neuroendocrine markers chromogranin and synaptophysin. She recovered uneventfully and was kept on follow-up.

**Figure 1:**
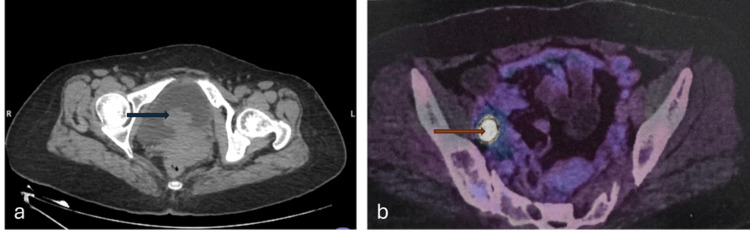
CECT and DOTANOC PET/CT findings (A) CECT scan showing a broad-based polypoid macrolobulated arterial-enhancing intraluminal mass lesion, measuring 3.6×2.9×2.2 cm (CC×AP×TS), epicentered in the postero-inferior wall of the urinary bladder (blue arrow). (B) DOTANOC PET/CT showing the appearance of ^68^Ga-DOTANOC avid right external iliac node (orange arrow), measuring ~ 13×10 mm (SUV max ~71). CECT: contrast-enhanced computed tomography; CC: craniocaudal; AP: anteroposterior; TS: transverse section; PET: positron emission tomography; CT: computed tomography; SUV: standardized uptake value

Six months later, she developed recurrent hematuria accompanied by palpitations and episodic diaphoresis. Blood pressure (BP) recordings showed labile hypertension. Biochemical evaluation confirmed markedly elevated urinary normetanephrines (3239 μg/24 h; normal 119-451 μg/24 h). ^68^Ga-DOTANOC PET/CT demonstrated a recurrent lobulated posterior bladder wall mass with avid uptake and an avid right external iliac lymph node, consistent with local recurrence and metastasis (Figure 1). She was commenced on α-blockade with prazosin, followed by β-blockade with metoprolol for hemodynamic stabilization, along with preoperative salt and fluid loading.

**Figure 2:**
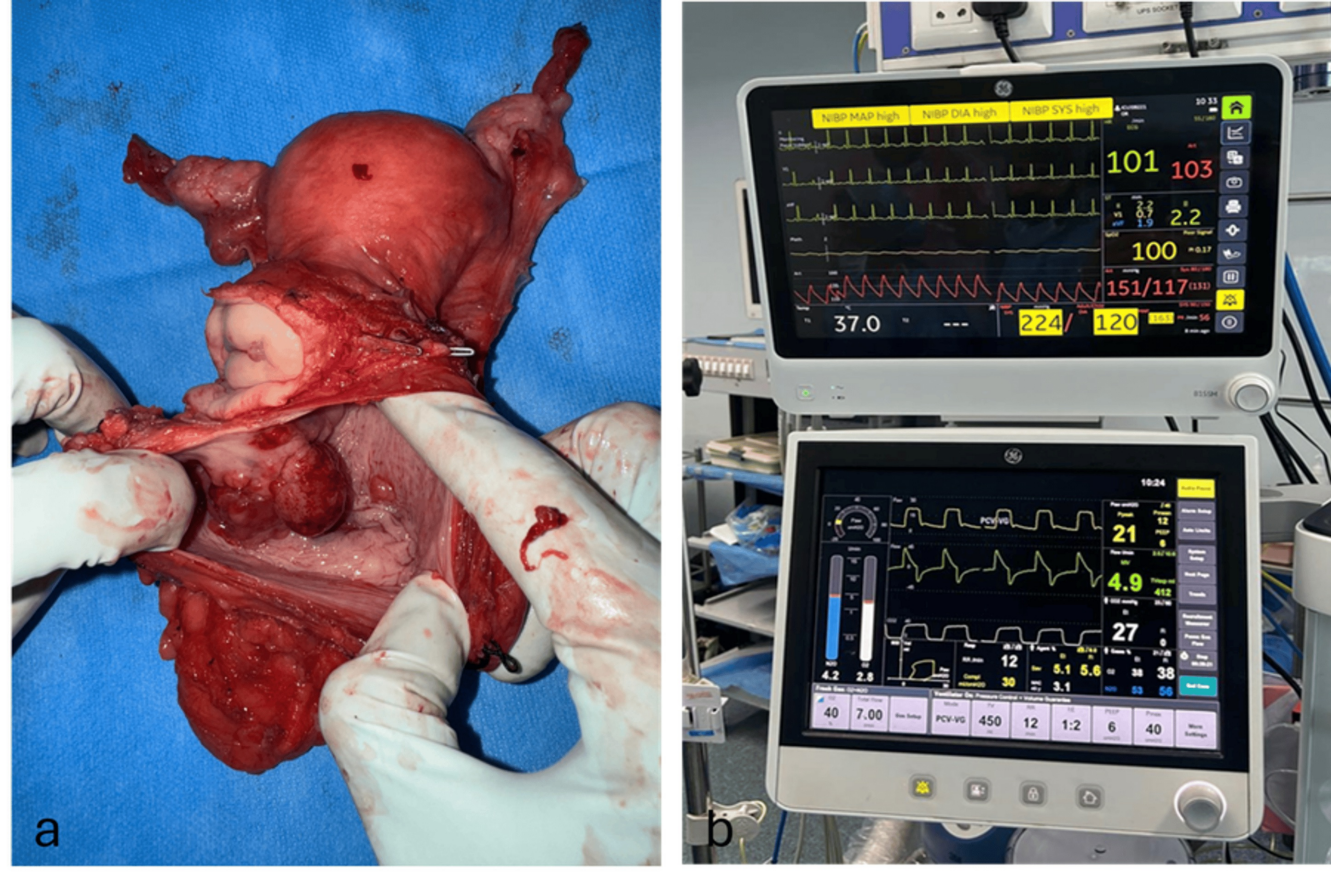
Resected surgical specimen of the malignant bladder paraganglioma and intraoperative anaesthetic monitor recording during pelvic exenteration (A) Gross photograph of the anterior pelvic exenteration specimen showing the urinary bladder with an intramural posterior wall tumor. The lesion appears lobulated and infiltrative, consistent with malignant paraganglioma. Surrounding perivesical fat and adjacent structures were removed en bloc as part of the oncologic resection. (B) Anaesthetic monitor showing a hypertensive crisis with blood pressure spike to 224/120 mmHg during anterior pelvic exenteration for malignant bladder paraganglioma, consistent with catecholamine surge. The heart rate was 101/min with preserved oxygen saturation and stable ventilation parameters.

Definitive surgery with anterior pelvic exenteration and ileal conduit diversion was performed (Figure 2A). During bladder mobilization, she developed a hypertensive crisis with BP of 224/120 mmHg (Figure 2B), controlled with intravenous nitroglycerin infusion. The procedure was completed successfully, and in the immediate postoperative period, she required temporary adrenaline support in the intensive care unit before stabilizing and recovering uneventfully.

**Figure 3:**
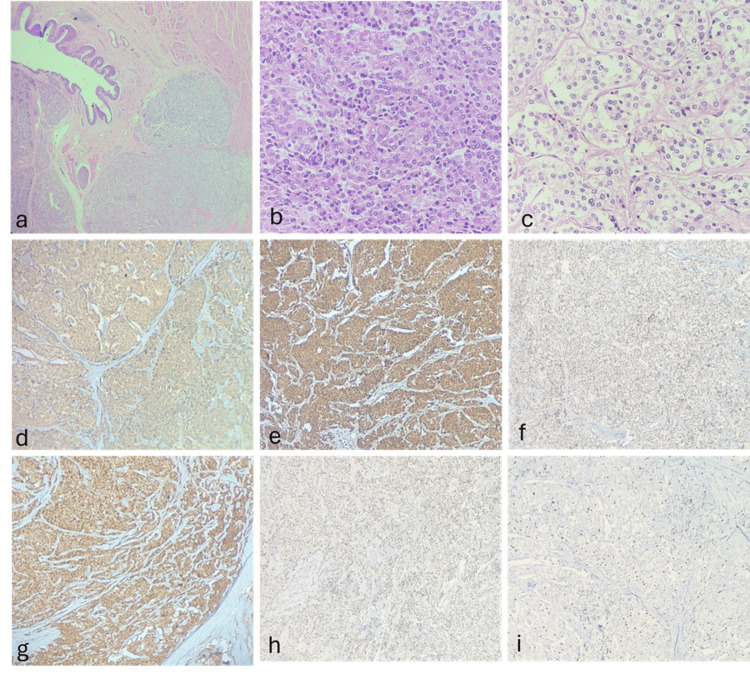
Histopathology and immunohistochemistry of the malignant bladder paraganglioma (A) Low-power view (H&E, ×4) showing the urinary bladder urothelial lining with the underlying subepithelium infiltrated by tumor cells arranged in nests, islands, and sheets. (B) High-power view (H&E, ×100) demonstrating the round to polygonal tumor cells with eosinophilic to clear cytoplasm, round nuclei, and characteristic salt-and-pepper chromatin. (C) High-power view (H&E, ×400) showing the tumor cells arranged in sheets and islands with intervening fibrovascular stroma. (D) Strong cytoplasmic expression of synaptophysin. (E) Diffuse positivity for chromogranin. (F) Focal nuclear positivity for GATA3. (G) S100 highlighting sustentacular cells. (H) Strong nuclear staining for INSM1. (I) Ki-67 proliferation index of 40%, confirming high-grade aggressive behavior. H&E: hematoxylin and eosin

Histopathological examination of the surgical specimen confirmed malignant extra-adrenal paraganglioma infiltrating perivesical fat (pT3b) with metastasis in one right external iliac lymph node (pN1). The tumor demonstrated large nests of cells with high cellularity (>250 cells/HPF), comedo-type necrosis, atypical mitoses, and vascular invasion. The GAPP score was 9, and the Ki-67 proliferation index was 40%, consistent with poorly differentiated high-risk paraganglioma. Immunohistochemistry confirmed neuroendocrine differentiation with positivity for synaptophysin, chromogranin, S100, and INSM1. Surgical margins were negative (Figure 3).

She was discharged in stable condition and continues six-monthly follow-up with urinary normetanephrines and cross-sectional imaging. At her most recent review, she remained asymptomatic and disease-free with preserved functional status (Table 1).

**Table 1:** Clinical course and management timeline Table showing sequential clinical events from initial hematuria, diagnosis, recurrence, preoperative optimization, radical surgery, and ongoing follow-up. CECT: contrast-enhanced computed tomography; TURBT: transurethral resection of the bladder tumor; PET: positron emission tomography

Month	Clinical events
Month -1	Painless hematuria
Month 0	CECT of the abdomen showed a broad-based polypoidal macrolobulated polypoidal arterial-enhancing intraluminal mass lesion of size 3.6×3 cm, epicentered in the posterior inferior wall of the urinary bladder
Month 0	TURBT
Month 6	Recurrent hematuria and palpitations
Month 6	DOTANOC PET: appearance of irregular lobulated soft tissue density mass lesion in the urinary bladder, with significant ^68^Ga-DOTANOC uptake, arising from the posterior wall, suggestive of disease recurrence. Appearance of ^68^Ga-DOTANOC avid right external iliac node, suggestive of nodal metastasis
Months 6-7	Antihypertensive measures (α- and β-blockade)
Month 8	Total anterior pelvic exenteration + ileal conduit performed
Month 9	Regular follow-up

## Discussion

Paragangliomas of the urinary bladder are exceedingly rare, constituting less than 0.05% of all bladder tumors. They are typically diagnosed between the fourth and fifth decades of life, with a slight female predominance. Clinically, macroscopic hematuria is the most frequent symptom, observed in more than 60% of cases, while adrenergic manifestations such as paroxysmal hypertension, palpitations, diaphoresis, and syncope occur in up to half, often precipitated by micturition. Nevertheless, a subset of patients may present without these classical features, as seen in our case, leading to diagnostic delay [6].

Biochemical assessment with plasma or urinary fractionated metanephrines is the most sensitive diagnostic approach. Marked elevation of normetanephrines in our patient confirmed catecholamine secretion, which contributes to both the symptom profile and perioperative hemodynamic instability. Therefore, preoperative α-adrenergic blockade remains an essential component of management to prevent intraoperative hypertensive crises.

Radiologically, CECT typically reveals a well-defined, hyperenhancing intramural bladder mass, and MRI demonstrates the characteristic "light-bulb bright" appearance on T2-weighted imaging [7]. Functional imaging has evolved substantially, although ^123^I-MIBG scintigraphy was the historical standard, ^68^Ga-DOTANOC PET/CT offers superior sensitivity for detecting both primary and metastatic lesions [8]. In the present case, PET/CT effectively delineated local recurrence and nodal involvement.

Histopathological examination classically shows a zellballen architecture comprising chief and sustentacular cells. Immunohistochemistry for chromogranin, synaptophysin, INSM1, and S100 confirms neuroendocrine differentiation. Malignancy is determined by evidence of invasion or metastasis rather than cytological atypia. The GAPP system aids in prognostic stratification, correlating with proliferative activity; our patient's high GAPP score (9) and Ki-67 index (40%) indicated poorly differentiated, aggressive behavior [9].

Surgical excision remains the cornerstone of therapy. Localized tumors may be managed by partial cystectomy or wide local excision, while TURBT is frequently inadequate as a definitive treatment, owing to the risk of incomplete tumor clearance. Radical cystectomy or anterior pelvic exenteration is reserved for locally advanced or recurrent disease. Adequate preoperative α-blockade (e.g., phenoxybenzamine or prazosin), followed by β-blockade if needed, is vital to prevent intraoperative catecholamine surges. Despite this, unpredictable hypertensive crises may still occur, as in our case.

Prognosis remains guarded in malignant bladder paraganglioma, with recurrence rates reported between 15% and 30% even after complete resection. Lymph node or distant metastasis confers a poor outcome. Lifelong follow-up with biochemical evaluation and cross-sectional or functional imaging at 6-12-month intervals is strongly recommended according to Endocrine Society guidelines [10].

## Conclusions

Bladder paragangliomas, though exceedingly rare, are tumors of profound clinical relevance. Their presentation can be deceptively indolent, often limited to hematuria, yet they possess the potential for catecholamine crises, local invasion, recurrence, and distant metastasis. Early recognition demands a high index of suspicion, guided by biochemical and functional imaging assessment. Radical surgery remains the therapeutic mainstay for malignant or recurrent disease, but safe operative outcomes hinge on meticulous preoperative adrenergic blockade. Owing to their unpredictable natural history, these tumors warrant vigilant, lifelong surveillance. A multidisciplinary approach integrating urology, endocrinology, pathology, and nuclear medicine remains essential for optimal management and long-term disease control.
